# Effect of Vitrectomy for Full-Thickness Macular Hole on Three-Dimensional Macular Shape

**DOI:** 10.1177/24741264251367112

**Published:** 2025-09-10

**Authors:** Stewart Lake, Keryn Williams, Murk Bottema, Karen Reynolds

**Affiliations:** 1 Medical Device Research Institute, College of Science & Engineering, Flinders University, Adelaide, SA, Australia; 2 Health & Medical Research Institute, College of Medicine & Public Health, Flinders University, Adelaide, SA, Australia

**Keywords:** macular hole, posterior vitreous detachment, optical coherence tomography, vitrectomy

## Abstract

**Purpose:** To examine whether vitrectomy surgery for macular hole alters the 3-dimensional shape of the macula. **Methods:** Macular shape was measured on radially oriented spectral-domain optical coherence tomography scans centered on the fovea. Eyes of patients with a macular hole were imaged before and after surgical repair. Eyes of individuals who had experienced a spontaneous posterior vitreous detachment (PVD) were also imaged. Pre- and postoperative shape was compared using the mean and range of best-fit curvature, and the difference between the best-fit curvature and retinal shape. **Results:** Fifty-two eyes of 17 men and 35 women with a macular hole (mean ± SD age, 69.2 ± 8.0 years) were studied. The mean (±SD) axial length was 23.84 ± 1.63 mm. A successful surgical outcome was achieved in 48 of the 52 eyes. Mean (±SD) macular curvature was significantly reduced, from 0.027 ± 0.016 mm^−1^ presurgery to 0.025 ± 0.015 mm^−1^ postsurgery (*P* = .01). There were no other significant differences in macular shape measurements between the pre- and postoperative states (all *P* > .05). No significant change in macular shape was observed in the 28 eyes scanned before and after spontaneous PVD. **Conclusions:** Surgery for macular hole produces a significant reduction in macular curvature, not seen in eyes that develop a spontaneous PVD. These findings support the hypothesis that a mechanical force acts broadly across the macula in macular hole and may have implications for the area of intervention required for surgical repair.

## Introduction

Macular holes are round, full-thickness neurosensory retinal defects centered on the fovea, the thinnest part of the posterior retina and the structure responsible for the central and highest acuity vision.^1^ Holes are thought to arise from predominantly anteroposterior vitreomacular traction occurring during a period of incomplete posterior vitreous detachment (PVD).^2^ Perifoveal posterior hyaloid separation with persistent attachment at the fovea leads to a full-thickness defect with radial then tangential displacement of the retinal tissue. Developing in the sixth to eighth decades of life, idiopathic macular holes are thought to be associated with a failure of normal PVD formation.^3^ Presenting with a subacute blurring of vision, untreated over three quarters will progress to vision loss severe enough to meet the World Health Organization criteria for blindness in the affected eye.^4,5^

More common in women than in men, most idiopathic macular holes respond very well to surgical intervention undertaken within 6 months of onset. Vitrectomy with induction of posterior hyaloid face detachment and peeling of the inner limiting membrane of the retina has a reported anatomic success rate of > 90%.^6,7^ The procedure is thought to produce hole closure by removing the centripetal force through PVD induction. Removing the inner limiting membrane and associated residual vitreous cortex on the retinal surface removes tangential traction from the posterior hyaloid face and takes away the most inelastic retinal layer, enabling the retinal tissues displaced laterally during hole formation to migrate back toward their original position.^8^

Prior work has investigated the geometry of the posterior pole in eyes with macular hole, reporting differences in the major retinal artery trajectory from the optic nerve, as visualized by color fundus photography, in patients with macular hole.^9^ Peripheral retinal shape, as measured by optical coherence tomography (OCT), is known to vary by axial length,^10^ and mid peripheral retinal shape was found to be different in a comparison between eyes with retinal detachment and eyes with PVD.^11,12^

Based on the hypothesis that vitreomacular forces are involved in macular hole formation, this study investigated how surgery for macular hole affected the posterior pole shape, as measured with OCT within a 3-dimensional space. As a comparison group, images were obtained from a series of patients for whom radial OCT scans were available from both before and after occurrence of a spontaneous PVD.

## Methods

### Subjects

Individuals with an idiopathic full-thickness macular hole who attended ophthalmology clinics in South Australia between February 2019 and November 2023 were invited to participate. Radial high-definition (HD) OCT scans centered in the fovea were obtained prior to surgery, and the same scan protocol was repeated after surgery once the intraocular gas had cleared and the macula was visible. Surgery in all cases involved an inner limiting membrane peel with a radius of >1 disc diameter. Intraocular gas typically takes up to 2 months to disperse and allow imaging of the macula after surgery. Postoperative imaging was performed after this interval, and within the schedules of normal clinical reviews, with some disruption during the COVID-19 pandemic. Axial length was measured using an IOLMaster 500 (Carl Zeiss Meditec) for the macular hole group but not the spontaneous PVD group.

### Imaging

**Figure 1.**
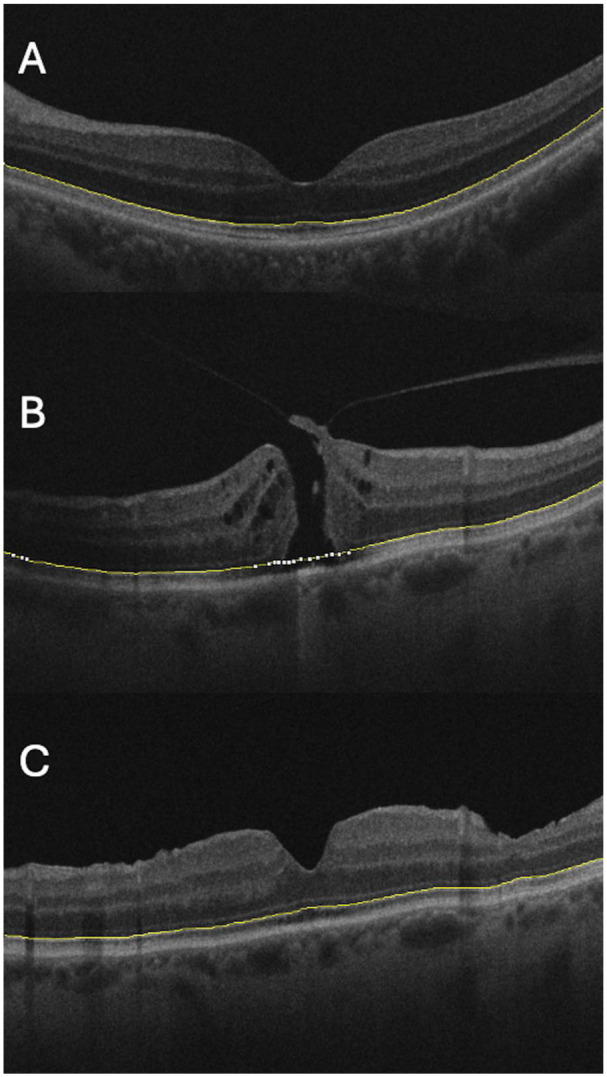
Sample optical coherence tomography (OCT) images. Retinal shape is identified by the yellow line. OCT images represent (A) a normal macula after posterior vitreous detachment, and a single macula (B) before vitrectomy surgery and (C) after surgery for full-thickness macular hole. Reduction in retinal curvature can be seen from pre- to postsurgery.

OCT imaging, using Cirrus spectral-domain OCT (Carl Zeiss Meditec), was performed with a single HD radial cube, acquiring 12 radially oriented B scans (each 6 mm), with each B scan rotated 15° incrementally around a central point at the fovea. Supplementary Video 1 illustrates the orientation of these B scans, showing a 3-dimensional video reconstruction of a macula. Retinal contour was determined from the OCT images using a Livewire plug-in^13^ for ImageJ (National Institutes of Health). Prior work has demonstrated the reproducibility of these measurements both between different graders and across repeated scans of the same eyes over time.^10,14^ The conventional marker for retinal shape on OCT has been the outer highly reflective band/retinal pigment epithelium. In our study, the high-intensity ellipsoid layer line was used to facilitate tracking. Where there were gaps, such as within the macular hole, the line was continued parallel to the retinal pigment epithelium. Examples of retinal shape identification from individual B scans are presented in Figure 1.

All data analyses were performed with MATLAB (The MathWorks Inc). The HD radial B scan acquisition protocol employs an identical raster pattern in both the right and left eye, which means that, for example, the scan oriented supero-temporal to infero nasal in the right eye will be oriented supero-nasal to infero temporal in the left eye. To account for this, left eye scans were reflected to match right eye scans.

### Image Analysis

The terms retinal shape and contour are used interchangeably herein, and refer to the path of the retinal pigment epithelium across the OCT B scan. The retinal contour in each OCT is measured across the 2 dimensions within the B scan, with the mean value for an eye obtained from 12 radial scans separated by 15° of rotation around an axial axis through the fovea. This provides a measure of the macula shape in 3 dimensions. Macular curvature was determined as the vertex curvature of the best-fit second-order quadratic curve fit to the retinal shape, calculated using the least squares method. Retinal irregularity was defined as the difference between the macular shape and the macular curvature, and analyzed in the frequency domain after fast Fourier transformation. Magnitude of irregularity was calculated in the frequency domain as the sum of the first 30 frequency bin moduli, with higher frequency bins discarded as noise.^10^ There were 12 curvature and 12 irregularity values for each eye, 1 of each per B scan. To manage the nested data structure, the shape of each eye was analyzed with mean and range of curvature and mean and range of irregularity.^15^ This is analogous to analysis of corneal shape, which is summarized as the average dioptric power and astigmatism (the difference between maximum and minimum power).

### Statistical Analysis

Comparisons of observations before and after surgery or PVD were performed with paired *t* tests. Two-sample *t* tests were performed to assess the significance of differences in secondary outcomes, including comparisons of the effects of sex on shape; to compare the effects of shape on surgical success; and to compare eyes with to eyes without a macular hole. Spearman’s rank-order correlation was used to investigate the relationship between axial length and change in curvature, and one-way ANOVA was used to estimate differences in change of curvature arising from the orientation of the B scan. Statistical significance was set at *P* ≤ .05. Mean values are ± SD.

The study was approved by the Southern Adelaide Local Health Network Human Research Ethics Committee and was performed in accordance with the tenets of the Declaration of Helsinki. Written informed consent was obtained from all participants.

## Results

Fifty-two patients with a macular hole (35 women, 17 men; 24 right eyes, 28 left eyes) had OCT imaging before and after surgery. Their mean age was 69.2 ± 8 years (range 51–90 years), and mean axial length was 23.84 ± 1.63 mm (range 21.88–30.24 mm). Forty-seven eyes had successful surgical outcomes (hole closure) with a single procedure, and 1 eye (a highly myopic eye) had a successful surgical outcome after a second procedure. The mean interval between first and final imaging was 5 ± 4 months.

Twenty-eight patients (11 men and 17 women; 28 eyes) were imaged both before and after the occurrence of a spontaneous PVD. Their mean age was 68.5 ± 7.3 years (range 53–82 years). Ten of the 28 patients were under review for medical retinal conditions, 12 for surgical conditions in the unaffected eye, and 3 for glaucoma (all reasons for attending the ophthalmology clinic prior to PVD occurrence are reported in Supplementary Table S1). A review post-PVD took place in order to evaluate the symptoms of PVD or to regularly monitor chronic disease, or both. The mean interval separating imaging before and after PVD was 10 ± 7 months.

**Figure 2.**
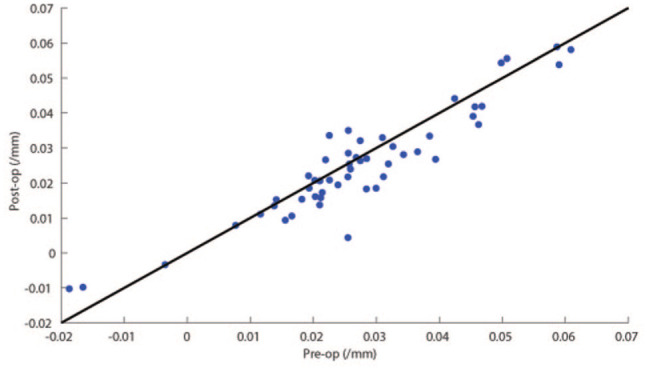
Scatterplot of postoperative versus preoperative curvature in eyes with macular hole. Each point represents the mean curvature of a single eye, with the line y = x equivalent to no change in curvature. There was a significant decrease in curvature after surgery (mean difference between pre- and postoperative curvature = 0.0021 mm; 95% CI, 0.0005 to 0.0040 mm; *P* = .01).

Measurements of the macula curvature and irregularity of eyes with macular hole are presented in Table 1. The mean macular curvature decreased significantly from before vitrectomy surgery (0.027 ± 0.016 mm^−1^) to after surgery (0.025 ± 0.015 mm^−1^; *P* = .01) (Figure 2). There were no significant differences in within-eye range of curvature or mean or range of irregularity between the pre- and postoperative states. There were no significant differences in any of the shape measurements between the sexes (*P* = .67–.99).

**Table 1.** Macular Curvature and Shape Irregularity Before and After Surgery for Macular Hole.

Macular Hole Eyes	Curvature^a^, mm^−1^		Shape Irregularity^a^, mm	
	K	Range	Irregularity	Range
Presurgery	0.027 (0.016)	0.016 (0.008)	3.040 (1.731)	2.601 (1.665)
Postsurgery	0.025 (0.015)	0.018 (0.010)	3.042 (1.655)	2.590 (1.651)
*P*, by *t*-test	.01	.08	.82	.91

a Values are the mean (SD).

Holes in 4 eyes failed to close postoperatively. There were no significant differences in mean shape between eyes with successful surgery outcomes (K_scs_) and those with unsuccessful surgery outcomes (K_fail_), with neither mean preoperative curvature (preoperative mean K_fail_ = 0.031, mean K_scs_ = 0.027, *P* = .66) nor mean postoperative curvature (postoperative mean K_fail_ = 0.028, mean K_scs_ = 0.025, *P* = .70) differing between successful and unsuccessful cases.

In analyses using one-way ANOVA to compare whether curvature change after macular hole surgery differed according to the orientation of the B scan, no differences in change in curvature were seen between B scan orientations (F_(df 11, 612)_ = 0.26, *P* = .99), implying that there was a high degree of radial uniformity in change of shape with surgery. No correlation (by Spearman’s rank correlation) was seen between the change in curvature and axial length (*P* = .38).

Table 2 presents the results of curvature and irregularity measurements for the spontaneous PVD group. There were 17 women and 11 men who had a spontaneous PVD. No significant shape change was seen in the 28 eyes imaged before spontaneous PVD and after spontaneous PVD, across any of the measures. In particular, no significant differences in mean curvature were seen in eyes before PVD and after PVD (before PVD, K = 0.022 ± 0.016 mm^−1^ versus after PVD, K = 0.021 ± 0.017 mm^−1^; *P* = .68). There was no significant difference between the preoperative curvature of eyes with macular hole and those eyes imaged prior to PVD (*P* = .15).

**Table 2.** Macular Curvature and Shape Irregularity Before and After Spontaneous PVD.

Spontaneous PVD Eyes	Curvature^a^, mm^−1^		Shape Irregularity^a^, mm	
	K	Range	Irregularity	Range
Before PVD	0.022 (0.016)	0.023 (0.012)	3.822 (1.867)	3.550 (1.982)
After PVD	0.021 (0.017)	0.023 (0.014)	3.797 (1.722)	3.591 (1.981)
*P*, by *t* test	.68	.88	.81	.76

Abbreviation: PVD, posterior vitreous detachment.

a Values are the mean (SD).

## Conclusions

To our knowledge, this is the first report to identify a change in macular shape following macular hole surgery. The reduction in curvature that occurred after surgical posterior hyaloid separation was not seen in eyes with spontaneous PVD, suggesting that the change was related to either the existence of the macular hole, the surgery, or both. Statistical significance was achieved with the small absolute change in curvature by measurement of the same eyes before and after surgery. This enables the use of the more sensitive paired (as opposed to unpaired) *t* test. The lack of any curvature change differences between the successful and unsuccessful surgery groups (albeit with a small number of the latter) suggests that shape change does not correlate with surgery success. This study considered shape using the conventional measure of the retinal pigment epithelial line contour, indicating a change in anatomy has occurred across the full thickness of the retina, and not confined to the inner retinal structures alone. Retinal irregularity was found to be the same in both groups. This finding differentiates our study from previous work on shape in myopia and retinal detachment, where irregularity was more useful than curvature.^10,11^

If the curvature change from surgery were due solely to relief of radial traction confined to the central fovea, as opposed to relief of the broader macular area, it would be expected that curvature would increase in the postoperative period. The decline in curvature postsurgery implies that any force acts across a broader area than the fovea alone, with the shape change coming from force acting on the peripheral macular area. The high degree of uniformity in curvature change by scan orientation suggests that any mechanical effect is radially symmetrical. A more widespread extra-macular contraction affecting the mid peripheral and anterior retina might be another explanation for the increased posterior curvature. This mechanism could potentially produce a measurable increase in axial length, which has not been reported to date.

Another explanation for the change in shape with surgery is a direct effect of surgery on the shape of an eye. While cataract surgical incisions are known to alter corneal curvature, incisions for vitrectomy are smaller and further from the posterior pole than a corneal incision is to the cornea. Vitrectomy has not been shown to alter refraction and hence has little effect on the corneal shape, which is more proximal to the vitrectomy incisions than the incisions are to the macula.^16,17^ It therefore seems improbable that the surgical incisions influence posterior curvature. It is current standard of care to remove or separate the inner limiting membrane from part or all of the macula during surgery, after surgical induction of PVD.^8,18^ Our conclusion is that the change in shape relates to the pathophysiology of macular hole and the surgical release of traction across the broader macula and mid peripheral retina.

Eyes with macular hole had a greater mean curvature before and after surgery compared with the spontaneous PVD group, although there was no statistically significant difference between the diagnostic groups. This work reports on shape change after spontaneous PVD in 28 patients, which was assessed despite the practical difficulty in capturing images of eyes before as well as after the PVD. Differences between the spontaneous PVD and macular hole groups included a greater time interval between the 2 observations in the spontaneous PVD cohort compared to the surgery cohort. Information on the axial length of the eyes with spontaneous PVD was not available, but our results indicate that there was no correlation between curvature change and axial length within the macular hole group. It would require an observation of shape prior to macular hole formation, and then again once the hole has occurred, to demonstrate that curvature increases with development of full-thickness macular hole. Such data are unavailable at this time.

Eyes with macular hole have been reported to have a greater retina artery trajectory^9^ and wider foveal base in the affected eye compared with the unaffected eye,^19^ both of which were interpreted as evidence of tangential force effects on the retina remote from the fovea. Those reports described differences in anatomy in a 2-dimensional space and compared eyes with and eyes without pathology. This is, to our knowledge, the first report to document differences in shape in 3 dimensions, and differences in shape seen before and after closure of the macular hole. This work provides more evidence to support the hypothesis that macular hole formation involves a process of mechanical force displacing the retina across a broader area than the fovea alone, and that the therapeutic effect of careful PVD induction is an essential component of the management of macular hole.

## Supplemental Material

sj-docx-1-vrd-10.1177_24741264251367112 – Supplemental material for Effect of Vitrectomy for Full Thickness Macular Hole on Three-Dimensional Macular Shape Supplemental material, sj-docx-1-vrd-10.1177_24741264251367112 for Effect of Vitrectomy for Full Thickness Macular Hole on Three-Dimensional Macular Shape by Stewart Lake, Keryn Williams, Murk Bottema and Karen Reynolds in Journal of VitreoRetinal Diseases
