# Supplementary material for: Effect of Vitrectomy for Full Thickness Macular Hole on Three-Dimensional Macular Shape
Source: J Vitreoretin Dis. 2025 Sep 10:24741264251367112. Online ahead of print. doi: 10.1177/24741264251367112 (PMC12423084; doi:10.1177/24741264251367112)
Supplement: sj-docx-1-vrd-10.1177_24741264251367112 – Supplemental material for Effect of Vitrectomy for Full Thickness Macular Hole on Three-Dimensional Macular Shape [file sj-docx-1-vrd-10.1177_24741264251367112.docx]

Table S1. Reason that patients were attending the ophthalmology clinic, PVD group eyes.

| **Reason** | **Number** | **Comments** |
| --- | --- | --- |
| Monitoring of diabetes (no maculopathy) | 5 |  |
| Age related maculopathy | 4 | No macular contour disruption (hard drusen) |
| Glaucoma | 3 |  |
| Retinal detachment other eye | 3 |  |
| Cataract surgery other eye | 3 |  |
| Retinal tear other eye | 3 |  |
| VMT or ERM other eye | 2 |  |
| PVD other eye | 1 |  |
| Marfan’s syndrome | 1 |  |
| Retinal vein occlusion other eye | 1 |  |
| Macular hole other eye | 1 |  |
| Eye health check NAD | 1 |  |

VMT = vitreo-macular traction, ERM = epiretinal membrane, NAD = no abnormality found.
